# Cellular metabolism in Th9, Th17, and Treg cell differentiation

**DOI:** 10.1093/intimm/dxaf032

**Published:** 2025-06-09

**Authors:** Toshio Kanno, Keiko Nakano, Yusuke Endo

**Affiliations:** Department of Frontier Research and Development, Laboratory of Medical Omics Research, Kazusa DNA Research Institute, 2-6-7 Kazusa Kamatari, Kisarazu, Chiba 292-0818, Japan; Department of Frontier Research and Development, Laboratory of Medical Omics Research, Kazusa DNA Research Institute, 2-6-7 Kazusa Kamatari, Kisarazu, Chiba 292-0818, Japan; Department of Frontier Research and Development, Laboratory of Medical Omics Research, Kazusa DNA Research Institute, 2-6-7 Kazusa Kamatari, Kisarazu, Chiba 292-0818, Japan

**Keywords:** ACC1, lipid metabolism, RORγt

## Abstract

CD4^+^ helper T (Th) cell subsets play an essential role in the regulation of adaptive immunity. Th9, Th17, and regulatory T (Treg) cells require transforming growth factor-beta (TGF-β) for their differentiation; however, their respective functions are highly distinct. Recent studies have highlighted the critical role of cellular metabolism in initiating clonal expansion and facilitating the effector differentiation of Th cells. Upon antigen exposure, naïve CD4^+^ T cells undergo metabolic reprogramming to fulfill their bioenergetic and biosynthetic demands. This process involves a shift from fatty acid oxidation to glycolysis, which ensures a sufficient energy supply for activation and proliferation. Lipid metabolism plays a pivotal role in modulating the differentiation and function of Th17, Treg, and Th9 cells. This review explores the influence of metabolic pathways on key transcription factors, including retinoic-acid-related orphan receptor gamma t (RORγt) and SMADs, and emphasizes their regulatory roles in Th cell differentiation. Furthermore, it discusses emerging therapeutic strategies aimed at targeting cellular metabolism to address autoimmune and inflammatory diseases associated with these T-cell subsets.

## Teaser

Lipid metabolism in Th17, Treg and Th9 cells

## Introduction

CD4^+^ T helper 17 (Th17) cells are an effector lineage that differentiates from naïve T cells under the influence of specific cytokine signals (1, 2). Interleukin-6 (IL-6) and IL-21, together with transforming growth factor-β (TGF-β) and IL-1β, activate STAT3 and synergistically drive the expression of the Th17 master regulator, retinoic-acid-related orphan receptor gamma t (RORγt) (3, 4). RORγt is a lineage-defining nuclear receptor transcription factor that directly controls Th17 signature genes, such as *IL17A*, *IL17F*, *IL21*, and *IL23R* (5). In the presence of TGF-β alone, naïve T cells upregulate the transcription factor Foxp3, yielding induced regulatory T (iTreg) cells (6). Notably, when TGF-β is combined with IL-6 (or IL-21), both RORγt and Foxp3 are transiently induced, but the suppressive function of Foxp3 is antagonized by IL-6 signaling, allowing RORγt^+^ cells to develop into Th17 cells (7). Foxp3 and RORγt can physically interact in these intermediate cells, with Foxp3 dominantly repressing RORγt, unless pro-inflammatory cytokines (IL-6/IL-21) relieve this repression (8, 9). Thus, cytokine signals and transcription factor networks orchestrate the Th17/Treg cell fate decision, wherein RORγt is pivotal for committing to the Th17 lineage. Th17 cells are highly pro-inflammatory and have been implicated as pathogenic mediators in multiple autoimmune diseases (e.g. multiple sclerosis, rheumatoid arthritis, and psoriasis), making understanding their regulation critical (10–12). Similar to Th17 cells and Tregs, Th9 cells that differentiate from naïve CD4^+^ T cells in the presence of IL-4 and TGF-β were identified (13). Th9 cells appear to be a T-cell subset specialized for IL-9 production, in contrast to other subsets, such as Th17 cells and Tregs, which produce only small amounts of IL-9 (14). Th9 cells exhibit pro-inflammatory activity that mediates allergic inflammation and antitumor immunity (15). Recent findings have indicated that Th9 and Treg cells act in an opposing manner to regulate allergic and tumor-specific immune responses (16, 17). Several experiments have demonstrated that the imbalance between Th9 cells and Treg cells is closely associated with the pathogenesis of asthma and cancer (18–20).

**Figure 1.**
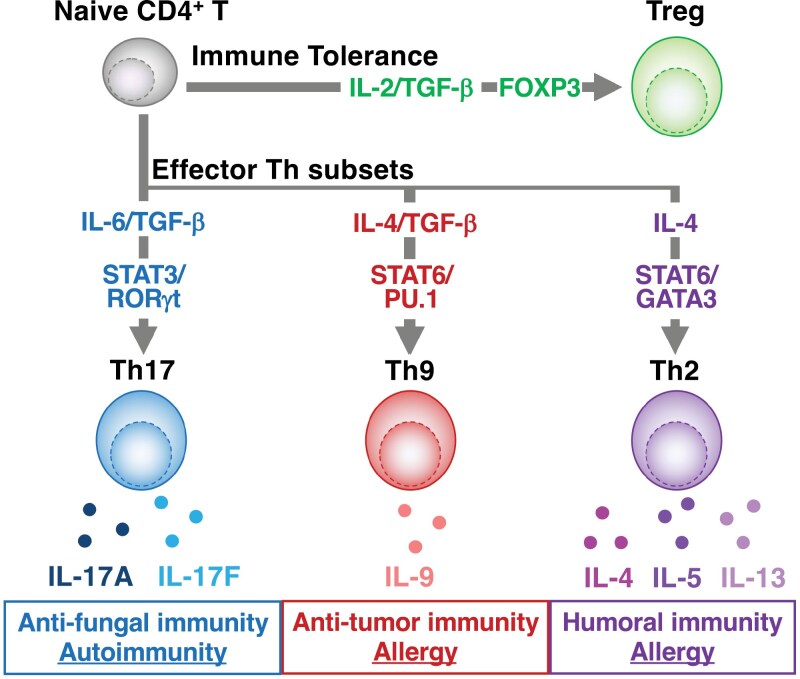
Naïve CD4^+^ T-cells differentiate into effector Th-cell subsets. Upon activation, naïve CD4⁺ T cells differentiate into distinct effector T-helper cell subsets, including Th2, Th17, regulatory T (Treg), and Th9 cells, possessing specialized helper functions. Th2 cells differentiate in the presence of IL-4, resulting in the induction of STAT6/GATA3 expression and the production of IL-4, IL-5, and IL-13. The differentiation of Treg cells is heavily dependent on TGF-β and IL-2, which induce the transcription factor Foxp3. The differentiation of Th17 cells requires IL-6 and TGF-β to induce RORγt. RORγt is a master transcription factor that orchestrates Th17 signature genes such as *Il17*, Ccr6, and *Il23r*. Th9 cells are efficiently induced under the conditions of IL-4 and TGF-β.

In recent years, increasing evidence has highlighted the relationship between cellular metabolism and various aspects of T-cell biology, including their full activation and differentiation into specific subsets (21–26). To meet the high-energy demands required for activation and rapid proliferation, T cells undergo a metabolic shift from fatty acid β-oxidation (FAO) and catabolic metabolism to aerobic glycolysis and anabolic metabolism (27). In addition to ATP production, the accumulation and synthesis of metabolic intermediates play crucial roles in T-cell differentiation (23, 27). Additionally, there is a strong link between the specialized functions of Th-cell subsets and distinct metabolic pathways such as glycolysis, FAO, and lipid biosynthesis (22, 24, 28–30). Lipid metabolites play a pivotal role in cell structure formation and several cellular processes, including acting as signaling mediators, maintaining energy balance, and regulating protein tethering (22, 26–28, 30–34). These processes are essential for the function and regulation of Th17, Treg, and Th9-cell subsets, highlighting the fundamental connection between metabolism and immune cell functionality (**Fig. 1**).

This review explores recent findings on the influence of cellular metabolism on the differentiation and function of Th17, Treg, and Th9 cells. We first examined the role of metabolic reprogramming, particularly lipid metabolism, in shaping the differentiation of these Th-cell subsets. Additionally, we describe how cellular metabolism controls key transcription factors, such as SMADs, RORγt, and other nuclear receptors. Finally, we discuss future research directions and potential therapeutic applications of targeting cellular metabolism to treat inflammatory and autoimmune disorders associated with Th17, Treg, and Th9 cells.

## Glycolytic pathway and Th17-cell differentiation

The differentiation of Th17 cells is supported by a metabolic bias toward glycolysis and anabolic metabolism (29, 35, 36). Key growth regulators sense the environmental cues that modulate this metabolic program. Mammalian target of rapamycin (mTOR) complex 1 is activated by nutrient and cytokine signals and promotes glycolysis, partly by upregulating hypoxia-inducible factor 1α (HIF1α) (37). HIF1α is a metabolic sensor that drives the expression of Glut1 and glycolytic enzymes (e.g. PDK1) to funnel pyruvate away from the TCA cycle and into lactate production (38). Th17 cells highly express HIF1α and require it for the full effector function. HIF1α deficiency in T cells leads to reduced Th17 differentiation and enhanced Treg development (37). Indeed, blocking glycolysis with 2-deoxyglucose or mTOR inhibition (rapamycin) similarly skews differentiation away from Th17 and towards Foxp3^+^ Tregs (37, 39). Mechanistically, HIF1α acts as a metabolic switch in the Th17/Treg balance by transactivating RORγt target genes and concurrently binding to Foxp3, leading to its proteasomal degradation (40). This dual role ensures that under high glycolytic conditions, RORγt is stabilized while Foxp3 is suppressed, tipping the balance decisively toward Th17 lineage commitment. Th17 cells are consistently characterized by high glucose uptake and lactate production, whereas induction of a more oxidative metabolic state impairs their differentiation. Thus, glycolysis and HIF1α/mTOR signaling have emerged as critical drivers of Th17 cell development, linking environmental nutrient availability to inflammatory T-cell responses.

## AMP-activated protein kinase and oxidative metabolism promote Treg differentiation

In contrast to the glycolytic program of Th17 cells, Tregs favor oxidative metabolism and catabolic pathways (27, 41). AMP-activated protein kinase (AMPK) is a key energy sensor activated by an increased AMP/ATP ratio (low-energy status). AMPK inhibits anabolic processes and promotes catabolic pathways such as FAO and autophagy to restore energy balance (42, 43). TCR signaling can activate AMPK (*via* Ca^2+^ influx and LKB1 kinase), which then phosphorylates and inactivates acetyl-CoA carboxylase (ACC1/ACC2), thus blunting fatty acid synthesis and enhancing FAO (42, 43). Inducible Tregs have higher AMPK activity and predominantly rely on FAO (rather than glycolysis) to meet their energy requirements (27). Pharmacological activation of AMPK promotes T-cell differentiation toward Tregs at the expense of Th17 cells (44–47). For example, the AMPK activator metformin (widely used in type 2 diabetes) increases Treg frequency *in vivo* and significantly reduces the production of IL-17 in patients and mouse models (44, 45, 47). By restraining mTOR activity, AMPK creates a metabolic environment unfavorable for Th17 development and supports Foxp3^+^ Treg stability (48, 49). Conversely, loss of AMPK activity results in hyperactive mTOR signaling and a propensity for inflammatory T-cell responses, although chronic AMPK deficiency can paradoxically impair the effector T-cell function because of metabolic exhaustion (50). In summary, AMPK-driven oxidative metabolism counterbalances mTOR/HIF1α-driven glycolysis, reinforcing Treg differentiation and immunosuppressive functions. The reciprocal metabolic profiles of Th17 and Treg cells, glycolysis, and fatty acid oxidation underscore metabolism as a decisive factor in lineage commitment.

## Lipid metabolism in Th17 cells and Treg cells

### Phospholipid metabolism

In addition to glucose, lipid metabolism plays a fundamental role in regulating the fate of Th cells. Activated T cells synthesize new lipids for cell membranes and signaling molecules, making *de novo* fatty acid synthesis a crucial process during clonal expansion (29, 35). Th17 cells, in particular, are highly dependent on endogenous fatty acid synthesis and show minimal use of exogenous fatty acids for proliferation and cytokine production (29, 35). Key lipogenic enzymes, ACC1 and fatty acid synthase (FASN), are upregulated as part of the Th17 metabolic program and represent the rate-limiting steps in fatty acid synthesis (51). Conversely, the TGF-β-SMAD2/3 pathway in Treg cells regulates FAO *via* the upregulation of fatty acyl-CoA synthetases, such as Acsl6 and Acsbg1, thereby controlling Treg cell differentiation (32). Acsbg1-mediated FAO is crucial for maintaining mitochondrial biogenesis in lung Treg cells and plays a key role in resolving lung inflammation (32). Consistently, exogenous oleic acid increased p-STAT5, upregulated the expression of FOXP3, and promoted OXPHOS in Treg cells (52). In contrast, in Th17 cells activated *in vitro*, a surge in fatty acid synthesis was observed alongside the suppression of FAO, suggesting a trade-off that favors building new lipids over burning them. Notably, the pharmacological inhibition of ACC1 or genetic deletion of ACC1 severely impairs Th17 cell differentiation (35, 53). Interestingly, fatty acid flux has also emerged as an important regulator of Th17/Treg plasticity (29, 35, 53). The pharmacological inhibition or genetic deletion of ACC1 increases the expression of the transcription factor FOXP3, which is a marker of Treg cells. The inhibition of Th17-cell generation caused by ACC1 inhibition can be rescued by supplementation with exogenous fatty acids, indicating that Th17 cells require a sufficient supply of lipids, but preferentially obtain them through internal synthesis. Furthermore, exogenous oleic acid has been reported to affect Th17 differentiation in CD4^+^ T cells (54). Upon activation, CD4^+^ T cells pre-exposed to oleic acid increased IL-17 production by upregulating genes encoding key enzymes responsible for cholesterol and fatty acid biosynthesis. *In vivo*, T-cell-specific ACC1 deletion or treatment with ACC inhibitors delayed the onset and reduced the severity of autoimmune disease in the EAE model, underlining the therapeutic relevance of targeting fatty acid metabolism. These findings reveal a metabolic checkpoint in which Th17 cells need to “make their own” fatty acids (*via* ACC1/FASN) to thrive, whereas Tregs can utilize environmental lipids and rely on FAO. Th17 cells play a pathological role in obesity and are largely dependent on ACC1 activity (29). Obesity induced by a high-fat diet promotes Th17 cell development and upregulates enzymes associated with fatty acid metabolism including ACC1 (29). CD4^+^ T cells from peripheral blood mononuclear cells (PBMCs) of obese individuals exhibit increased production of IL-17A and the expression of ACC1 (29, 55). Moreover, the frequency of IL-17A-producing CD4^+^ T cells is strongly correlated with the expression of ACC1 in obese patients (29). Studies have suggested that the increase in IL-17A-producing cells in obesity is driven by elevated IL-6 and IL-1β levels in the spleen and lungs, respectively (56, 57). These pro-inflammatory cytokines may influence the expression of genes related to fatty acid biosynthesis, including ACC1, in peripheral CD4^+^ T cells under obese conditions. This suggests a critical link between metabolic changes and immune responses in obesity, highlighting the potential role of ACC1 in obesity-associated inflammation and metabolic dysfunctions.

Mechanistically, lipid metabolism controls the activation of RORγt, a master transcription factor involved in Th17-cell differentiation in both mice and humans. Previous studies have established that RORγt plays a crucial role in the Th17 cell function, and its interaction with the coactivator p300 is necessary for its transcriptional activity (29). This study demonstrates that inhibition of ACC1, either genetically or pharmacologically, disrupts the nuclear colocalization of RORγt and p300 without affecting the expression of RORγt (29). The study further revealed that supplementation with oleic acid, a monounsaturated fatty acid (MUFA), restores Th17-cell differentiation and the nuclear interaction between RORγt and p300 (29). However, other fatty acids, such as saturated and polyunsaturated fatty acids (PUFAs), do not have the same effect. Interestingly, petroselinic acid, another MUFA, partially restored the Th17 phenotype. Using CRISPR-based screening, this study identified five lipid metabolic enzymes (*Scd2*, *Gpam*, *Gpat3*, *Lplat1*, and *Pla2g12a*) as crucial regulators of RORγt activation and Th17 gene expression (58). A lipidome analysis further identified 1-oleoyl-lysophosphatidylethanolamine (LPE [18:1]) as a critical metabolite that binds RORγt and acts as a physiological ligand. Deletion of *Pla2g12a* reduces LPE levels, impairing Th17 cell differentiation, whereas *Pla2g12a*-deficient mice show improved EAE pathology. These findings suggest that LPE (1-18:1) is a key modulator of the RORγt function and a potential therapeutic target for Th17-related autoimmune diseases.

### Cholesterol metabolism

Cholesterol metabolism also plays a significant role in regulating the Th17 cell functions (59, 60). Liver X receptors (LXRα/β), cholesterol-sensing nuclear receptors, help maintain the intracellular cholesterol balance by promoting cholesterol efflux *via* transporters ABCA1/G1 and suppressing lipid synthesis through the inhibition of SREBP-1c, a key regulator of lipogenic genes (28). Activation of LXR in T cells inhibits Th17 differentiation *in vitro* and reduces autoimmune disease severity *in vivo*, as evidenced by the fact that LXR agonists reduce Th17-cell frequencies and mitigate EAE pathology (61, 62). This inhibitory effect was reversed by the addition of mevalonate, a cholesterol biosynthesis precursor, indicating a direct role for cholesterol or its derivatives in Th17 development (62). Similarly, peroxisome proliferator-activated receptor-gamma (PPARγ), a lipid-activated transcription factor, suppresses Th17 differentiation by counteracting STAT3 activity and downregulating the expression of RORγt (63–65). Since RORγt is a lipid-binding nuclear receptor, specific oxysterols or cholesterol derivatives may influence Th17 cell activity.

### Ceramide/sphingolipid metabolism

Recent studies have highlighted the roles of sphingolipid and glycosphingolipid biosynthesis in the regulation of Th17 and Treg-cell differentiation (66–68). Through the integration of lipidomic and proteomic analyses, substantial alterations in both the quantity and composition of cellular lipids across the different effector Th-cell subsets were detected. Notably, the sphingolipid biosynthetic pathway is activated in Th1, Th2, Th17, and iTreg cells (66). Furthermore, pharmacological inhibition or genetic deletion of the rate-limiting enzymes serine palmitoyltransferase (SPT) and glucosylceramide synthase (GCS), which are key enzymes for the biosynthesis of sphingolipids from ceramide, abrogate the differentiation of Th17 cells and iTreg cells. Intriguingly, this was not observed in the Th0, Th1, and Th2 cells. Another study integrated genome-scale metabolic modeling (GSMM), lipidomic profiling, and *in vitro* gene silencing approaches to demonstrate the role of sphingolipids and glycosphingolipids in the differentiation of human Th17 and iTreg cells (67). Their findings further revealed that the composition of these lipids varied among different CD4^+^ Th-cell subsets (67). Specifically, in Th17 cells, the *de novo* sphingolipid pathway regulates the expression of IL-17A and IL-17F. Furthermore, Abimannan *et al*. found that *Sptlc1* deficiency increased intracellular reactive oxygen species (ROS), inhibited mTORC1 activity, led to the reduced expression of genes related to HIF-1α and the c-Myc-mediated glycolytic pathway, and suppressed Th17 differentiation (68). Consistent with this finding, *Sptlc1*^fl/fl^*CD4-*Cre mice showed attenuated severity of EAE and colitis. Collectively, these findings suggest that ceramide and the *de novo* sphingolipid biosynthetic pathway play pivotal roles in modulating Th17 and Treg differentiation.

**Figure 2.**
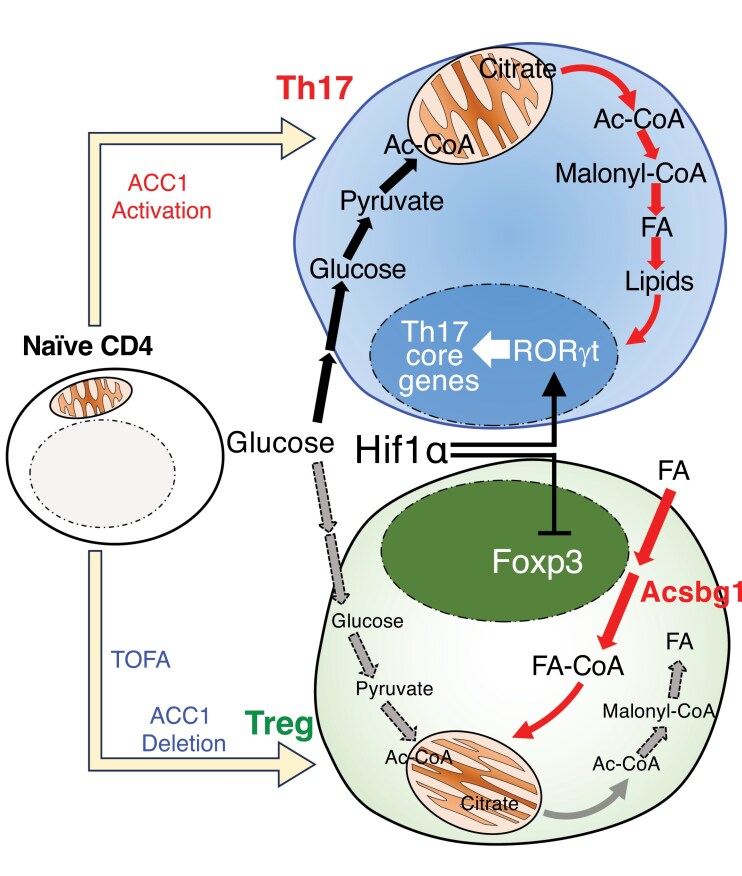
Metabolic control of Th17/Treg differentiation. Th17-cell differentiation heavily depends on *de novo* fatty acid (FA) biosynthesis. In CD4^+^ T cells, ACC1 deficiency significantly impairs Th17 differentiation. Pharmacological inhibition or genetic deletion of ACC1 increases Foxp3 expression cultured under Th17-skewing conditions. This shift towards Treg cell differentiation in ACC1-deficient cells could be overcome by the extrinsic supplementation of exogenous FAs. Additionally, Hif1α, a key regulator of glycolysis, distinctly influences Th17 and Treg differentiation. Thus, metabolic pathways play a crucial role in regulating the Th17/Treg balance.

In summary, Th17 and Treg-cell differentiation is governed by an intricate network of cytokine signals and transcriptional regulators that are inextricably linked to the cellular metabolic state (**Fig. 2**). Glycolysis and lipid synthesis feed the biosynthetic and signaling demands of Th17 cells, reinforced by factors such as HIF1α, mTOR, and RORγt, whereas oxidative metabolism and AMPK activity favor the Foxp3^+^ Treg lineage. Crosstalk between metabolic pathways and immune signaling is a critical determinant of the T-cell fate and function, and dysregulation of this crosstalk can contribute to autoimmunity. Thus, a detailed understanding of T-cell metabolism opens new avenues for immunomodulatory therapies, wherein metabolic reprogramming can be harnessed to selectively dampen pathogenic Th17 responses and potentially treat autoimmune diseases. The emerging field of immunometabolism continues to uncover how manipulation of metabolic checkpoints can restore immune homeostasis in diseases.

## Metabolic pathways in Th9-cell functions

### The multifaceted role of Th9 cells

In the presence of IL-4 and TGF-β, naïve CD4 T cells differentiate into Th9 cells, which are another distinct Th-cell subset characterized by IL-9 production. Murine models have revealed that IL-9 contributes to the development of allergic diseases and host defense against helminth and protozoan infections. IL-9 promotes the recruitment of monocytes, which differentiate into CD11c^+^ and CD11c^−^ interstitial macrophages, and these macrophages are essential for IL-9-dependent allergic responses (69). IL-9R-deficient mice also exhibit decreased lung ILC2 numbers, leading to impaired lung repair in the chronic phase after helminth-induced lung injury (70). Moreover, increasing evidence indicates that Th9 cells may have a potent ability to eradicate advanced tumors, particularly melanomas (16). Th9 cells elicit strong host antitumor CD8^+^ cytotoxic T lymphocyte responses by promoting Ccl20/Ccr6-dependent recruitment of dendritic cells to tumor tissues *via* the production of IL-9 (71). Signaling molecules and transcription factors downstream of TGF-β, IL-4, and IL-2 have been well studied in Th9 subsets. Impairment of the TGF-β receptor (TGF-βR), IL-4R, or IL-2R dampens the generation of Th9 cells. TGF-β signaling elicits SMAD-dependent and SMAD-independent signaling, thereby coordinately regulating Th9 differentiation. For example, phospho-SMAD2 or SMAD3 directly binds to the *Il9* gene locus. Deletion of *Smad2* and *Smad3* strongly decreases IL-9 secretion, leading to the improvement of OVA-induced airway inflammation (15, 72, 73). SMAD2 and SMAD3 cooperate with IRF4 to produce IL-9 in Th9 cells (72). SMAD4 also contributes to the regulation of the chromatin structure of the *Il9* locus, as well as SMAD2 and SMAD3 (74). The SMAD-independent TGF-β signaling pathway also involves other mitogen-activated protein kinases (MAPKs), including extracellular signal-regulated kinases 1/2, c-Jun N-terminal kinases, p38 MAPKs, phosphatidylinositol-3-kinase (PI3K)/protein kinase B (Akt, Rho-like GTPases, and protein phosphatase 2 A) (75). TAK1, a TGF-β-activated kinase, is an important mediator of the TGF-β-triggered SMAD-independent pathway (13, 76, 77). TAK1 activation suppresses the expression of Id3, which negatively regulates the expression of IL-9. PU.1 is also known to regulate Th9-associated genes. Since the mRNA expression of *Sfpi1*, which encodes PU.1, is still observed in SMAD-deficient T cells, PU.1 may be regulated by TGF-β in an SMAD-independent manner.

### Cellular metabolism contributing to the differentiation of Th9 cells

Accumulating evidence has demonstrated that cellular metabolism regulates the functions of Th9 cells as well as conventional Th subsets. Especially, Bertschi *et al*. have investigated the differences in cellular metabolism among Th1, Th2, and Th9 cells. *In vitro*-differentiated Th9 cells show higher protein expression of PPARγ, which is a classical ligand-activated nuclear receptor for the regulation of lipid and glucose metabolism, relative to Th1 or Th2 cells (78). While Th2 cells increase PPARγ expression compared to Th1 cells, TGF-β stimulation, which is required to Th9 cell differentiation, further enhances PPARγ expression. In the resting state, Th2 and Th9 cells display a similar level of glycolytic capacity, which is higher than that of Th1 cells. However, upon αCD3/αCD28 stimulation, glycolytic capacity is more strongly enhanced in Th9 cells than in Th1 or Th2 cells. Additionally, glucose uptake is greater in Th9 cells compared to Th1 or Th2 cells during TCR stimulation. Since the genes related to glycolysis are suppressed by PPARγ inhibition in Th9 cells, the augmented expression of PPARγ sustains higher glycolytic activity in Th9 cells. Consistently, IL-9 production was suppressed by the inhibition of glycolysis or by treatment with GW9662, a potent PPARγ antagonist. As observed in murine Th9 cells, human Th9 cells also showed enhanced glucose incorporation and glycolytic activity in comparison to hTh1 or hTh2 cells. The IL-9 production in hTh9 cells was also enhanced by extracellular glucose treatment in a dose-dependent manner. IL-9 was initially thought to be a Th2 cytokine because increased IL-9 production was observed alongside IL-4, IL-5, and IL-13 in animal models of nematode infection and allergic inflammation (79). IL-9-producing hTh cells share a chemokine receptor profile with CXCR3^−^CCR4^+^CCR6^−^ hTh2 cells. CXCR3^−^CCR4^+^CCR6^−^CCR8^+^ hTh9 cells secrete comparable levels of IL-4, IL-5, and IL-13 as CXCR3^−^CCR4^+^CCR6^−^ hTh2 cells. The downregulation of PPARγ by siRNA treatment moderately decreased IL-9 production. Although the activation of PPARγ results in the upregulation of glycolysis-related genes and activation of mTOR signaling, it remains unclear whether PPARγ directly or indirectly regulates the expression of these genes and signaling pathways. Another glycolysis-regulating factor is HIF-1α. Similar to its role in Th17 and Treg cells, HIF-1α also regulates Th9-cell differentiation by controlling glucose metabolism. The induction of hypoxia or genetic ablation of PHD2, which destabilizes the expression of HIF-1α protein, leads to the augmentation of IL-9 (17). As the expression of HIF1-α and IL-9 relies on EGFR signaling, EGFR-Hif1α signaling is important for the induction of IL-9 production (17). ATP-mediated nitric oxide (NO) is also known to activate HIF1α signaling *via* the nutrient sensor mTOR signaling pathway in Th9 cells. Indeed, blocking the P2X and P2Y P2-purinergic receptors, which recognize extracellular ATP, inhibits the differentiation of Th9 cells (80). SIRT1 is a highly conserved mammalian NAD^+^-dependent histone deacetylase that has emerged as a key metabolic sensor in various metabolic tissues (18). The ectopic expression of SIRT1 in CD4^+^ T cells inhibits IL-9 production and glycolysis. In contrast, *Sirt1*-deficient Th9 cells produce high levels of IL-9 by augmenting glycolytic activity. Intracellular pH is also important for the regulation of Th9 cells. Na^+^/H^+^ exchanger 1 (NHE1; gene name *Slc9α1*) is critically important for regulating intracellular pH by exporting H^+^ from the cell. Targeted NHE1 knockdown moderately decreases IL-9 production (81). Thus, several studies have highlighted the importance of glycolysis in the Th9-cell function. It has also been reported that the suppression of cellular metabolism enhances IL-9 production. Retinoic acid receptor α (RARα) belongs to the nuclear receptor family and is activated by retinoid acid (RA), an active derivative of Vitamin A (19). RA treatment strongly repressed IL-9 production in murine and human Th9 cells. Mechanistically, RA signaling has a major impact on Th9 cells by suppressing the global Th9 epigenome, thereby inhibiting the Th9 transcriptional program. The genetic deletion of RARα exacerbates papain-induced airway inflammation in Th9 cells. The expression of RA target genes is dysregulated in skin from patients with nickel allergy and in CD4^+^ T cells from atopic individuals stimulated *in vitro* with house dust mite extract. Therefore, the dysregulation of RA signaling in Th9 cells may accelerate allergic inflammation in humans (19).

### The role of lipid metabolism in Th9-cell differentiation

**Figure 3.**
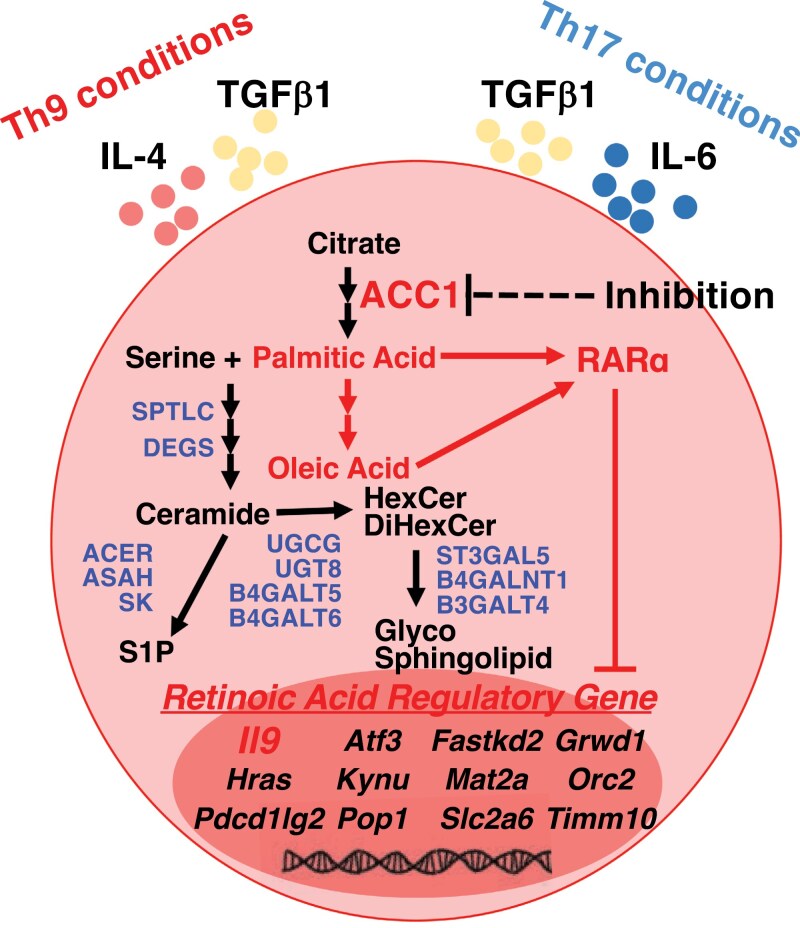
Lipid metabolism-mediated IL-9 production. Disruption of fatty acid metabolism enhances IL-9 production in both Th9 and Th17 cells. Inhibiting fatty acid metabolism decreases the amounts of oleic acid and palmitic acid, thereby leading to the suppression of RARα activity, which is a negative regulator of IL-9. In addition, the sphingolipid metabolic pathway is activated in Th17 cells. Since TGF-β1 is required for the generation of Th9 and Th17 cells, Th9 cells also may augment sphingolipid metabolism.

Interestingly, recent research has shown that fluctuations in fatty acid metabolism control the IL-9 production in murine and human Th9 cells (20). The suppression of ACC1, a fundamental enzyme in fatty acid biosynthesis, enhances the production of IL-9 in Th9 and Th17 cells. Furthermore, limiting extracellular fatty acids increases the production of IL-9 in these cells. ACC1 inhibition resulted in a permissive chromatin landscape at the *Il9* gene locus in Th9 and Th17 cells, as revealed by ATAC-seq and ChIP-seq. Mechanistically, defects in ACC1 activity disrupt RARα binding to its target locus, thereby impairing its suppressive function in IL-9 production. In addition, SMAD2/3-signaling is also essential for the augmentation of IL-9 production. A nontargeting lipidomic analysis revealed that 110 lipid species were commonly reduced in Th9 cells under fatty acid-limited conditions. The enhanced production of IL-9 is restored by supplementation with oleic acid or palmitic acid, which are the most and second-most abundant fatty acids, respectively, among the 110 commonly decreased lipids. Thus, lipid-containing oleic acid or palmitic acid may control the production of IL-9 in Th9 and Th17 cells. Consistent with this research, another study showed that the pharmacological inhibition of ACC1 increased the production of IL-9 in human Th9 cells (82). In addition to fatty acid metabolism, cholesterol metabolism negatively regulates the production of IL-9. Pharmacological inhibition or genetic deletion of *Hmgcr*, a rate-limiting enzyme of cholesterol biosynthesis, can enhance the differentiation of cytotoxic IL-9-producing cells (83). Furthermore, treatment with β-CD, which reduces the amount of cellular cholesterol, also increases the expression of IL-9. Mechanistically, cholesterol and its derivatives suppress the production of IL-9 by activating LXRs, leading to LXR sumoylation and reduced p65 binding to *Il9* promoter. Although some studies have conducted omics analyses to profile the transcripts, proteins, or metabolites in effector Th subsets, most of these studies focused on conventional Th subsets, including Th1, Th2, Th17, and Treg cells (66, 67). Few studies have investigated the lipidome profiles of Th9 cells. In human and mouse experiments, both Th17 and Treg cells shows distinct lipid profiles from Th1 and Th2 cells. These studies shows that metabolites of the sphingolipid pathway are enriched within Th17 and Treg cells. As TGF-β is required for the differentiation of Th17 and Treg cells, it may also contribute to the reprogramming of lipid metabolism. In mesangial cells, which are stromal cells that regulate kidney homeostasis, TGF-β increases the abundance of the mature form of SREBP-1 along with the induction of FAS protein. Adipocytes also directly upregulate the expression of TGF-β1 in response to feeding-linked insulin activity. Notably, the inhibition of TGF-β1 signaling also reduces adipose tissue mass. Thus, since Th9 cells require TGF-β1 for their differentiation, they likely exhibit a lipid profile that is more similar to that of Th17 and Treg cells than to that of Th1 or Th2 cells, including the accumulation of sphingolipid metabolites, in a TGF-β-dependent manner (**Fig. 3**).

## Conclusions

The regulation of lipid metabolism closely intersects with Th17-cell differentiation and function by influencing RORγt activation, particularly in pathological contexts. Therefore, elucidating the role of this metabolic pathway can shed light on potential novel therapeutic strategies for treating Th17-cell-mediated inflammatory diseases. In addition to Th17 cells, a recent study has highlighted the role of FA metabolism in Th9 cell differentiation. Reduced intracellular fatty acid levels promote Th9 cell differentiation, and inhibition of fatty acid biosynthesis in Th9 cells enhances their antitumor activity (20). The suppression of fatty acid synthesis appears to constrain the nuclear translocation of SMAD2/3 via the activation of RA-RARα signaling.

In summary, targeting Th17 or Th9 cells through the inhibitors affecting intracellular lipid metabolism is a cutting-edge approach to treating Th17-associated autoimmune disorders or augmenting Th9-mediated tumor rejection. However, achieving effective targeting necessitates a deeper comprehension of RORγt regulation in Th17 cells and other IL-17-producing immune cells during tissue inflammation. Moreover, it remains unclear whether there are variabilities in the efficacy of agents manipulating FA metabolism or inhibiting oxysterol synthesis across different autoimmune disorders. In addition, because Th9 cells have been studied less extensively than other CD4⁺ T-cell subsets, further investigation is needed to elucidate the mechanisms governing their differentiation. In particular, it is crucial to determine which specific intracellular fatty acid species contribute to the enhancement of their antitumor activity. Despite these challenges, targeting RORγt or Th9 cell program through cellular metabolism modulation will likely be pivotal in future autoimmune or antitumor therapy strategies.

## Funding

This work was supported by KAZUSA DNA Research Institute and grants from the Ministry of Education, Culture, Sports, Science and Technology of Japan (Grants-in-Aid: Grant-in-Aid for Early-Career Scientists 23K14552, and Scientific Research [B] 24K02246); Challenging Research (Exploratory) #24K22064; Transformative Research Areas (A) #25H01875; AMED-CREST (JP22gm1810002) from the Japan Agency for Medical Research and Development; FOREST (JPMJFR225X) from JST; TERUMO Life Science Foundation; Takeda Science Foundation; Mochida Memorial Foundation for Medical and Pharmaceutical Research; Uehara Memorial Foundation; Cell Science Research Foundation; Astellas Foundation for Research on Metabolic Disorders; MSD Life Science Foundation; Public Interest Incorporated Foundation; Canon Foundation; Princess Takamatsu Cancer Research Fund; Yasuda Medical Foundation; Mitsubishi Foundation; KOSE Cosmetology Research Foundation; Chemo-Sero-Therapeutic Research Institute; Toray Science Foundation; Shionogi Infectious Disease Research Promotion Foundation and Ono Pharmaceutical Foundation for Oncology, Immunology, and Neurology.
